# Child Stunting and Temperature Anomalies: A Cross-Sectional Study in Burkina Faso and Kenya

**DOI:** 10.3390/children12101346

**Published:** 2025-10-07

**Authors:** Tavis C. Mansfield, Molly E. Brown, Meredith L. Gore

**Affiliations:** Department of Geographical Sciences, University of Maryland, College Park 8200 Paint Branch Drive, College Park, MD 20742, USA; mbrown52@umd.edu (M.E.B.); gorem@umd.edu (M.L.G.)

**Keywords:** child malnutrition, Sub-Saharan Africa, multilevel modeling, heatwaves

## Abstract

Background/Objectives: Extreme temperatures linked to climate change threaten child health, particularly in Sub-Saharan Africa where malnutrition remains widespread. This study examines how exposure to hot and cold temperature anomalies influences child stunting in Burkina Faso and Kenya and evaluates how household infrastructure and socio-demographic factors interact with climate stressors to shape outcomes. Methods: We combined nationally representative Demographic and Health Surveys (Burkina Faso 2021; Kenya 2022) with daily maximum and minimum temperature data from the Climate Hazards InfraRed Temperature with Stations (CHIRTS). The analytic sample included children aged 24–59 months. Temperature anomalies were calculated as standardized deviations from local historical averages. Multilevel logistic regression models assessed associations between stunting, climate anomalies, and household-level factors, including electricity, water, sanitation, wealth, and rural/urban residence. Results: Heat anomalies were linked to increased stunting risk in Kenya (β = 2.34, *p* < 0.001), while in Burkina Faso, higher maximum temperatures unexpectedly reduced stunting odds (β = 0.08, *p* < 0.05). Cold anomalies showed marginal positive associations with stunting in both countries. Infrastructure and socioeconomic factors varied by context: electricity access and urban residence were protective in Burkina Faso, while improved sanitation, household wealth, and child sex differences were significant in Kenya. Conclusions: Climate anomalies and household conditions jointly influence stunting among children aged 24–59 months, with effects varying by country. Cold anomalies were associated with higher odds of stunting in Burkina Faso (BF OR = 2.14) and Kenya (KE OR = 1.20), while heat anomalies reduced stunting in BF (OR = 0.08) but increased it in KE (OR = 2.34). Electricity access was protective in both countries (BF OR = 0.61; KE OR = 0.71), while improved water, sanitation, and wealth were significant only in KE. Older child age consistently reduced stunting risk, and urban residence was protective only in BF. These findings underscore that climate impacts on stunting are context-specific and highlight the need for policies integrating climate adaptation with investments.

## 1. Introduction

Global climate change is projected to continue influencing the frequency and intensity of temperature shocks, including cold spells and heatwaves. While such events occur worldwide, their impacts are often more severe in Sub-Saharan Africa, where changing environmental conditions have rendered historically consistent climate patterns increasingly unpredictable [1,2]. Warming across the African continent is expected to exceed the global average, with projections indicating an increase of 3–6 °C by the end of the century under high-emissions scenarios and Sub-Saharan Africa identified as the most vulnerable region due to its limited capacity to adapt to or mitigate climate change [3]. Within this context, West and East Africa are projected to experience a rise in annual mean temperature of approximately 2 °C toward midcentury (2021–2050) under the RCP8.5 and SSP5-8.5 scenarios, surpassing the global average increase of about 1.5 °C, and to face more frequent and severe climate extremes, including droughts with significant social and environmental impacts [4,5]. Exposure to the current and predicted temperature extremes during childhood may lead to lasting health consequences, including impaired physical growth, cognitive development, and increased susceptibility to disease [6,7].

Stunting, or child undernutrition, is defined as low height-for-age and results from chronic or recurrent undernutrition, commonly associated with poverty, maternal malnutrition, frequent illness, and inadequate early-life feeding and care. Malnutrition during the first 1000 days can yield enduring repercussions on a child’s developmental trajectory [8], amplifying susceptibility to morbidity and mortality [9]. Anthropometric metrics commonly serve both as proxies for assessing individual child undernutrition and as an indicator of the food security status of a community [10,11]. Stunting, which is the focus of this paper, hampers children from reaching their full physical and cognitive potential and therefore has significant individual and social costs.

Exposure to temperature anomalies can impact child health through multiple interconnected pathways [12,13]. Studies indicate that exposure to cold indoor environments can negatively affect children’s health [14,15]. During cold weather, children’s bodies lose heat more rapidly than it can be generated, which depletes energy reserves and increases the risk of hypothermia, defined as a core body temperature below 95 °F (35 °C). Young children are particularly vulnerable because of their higher surface-area-to-body-mass ratio and limited ability to regulate body temperature [16]. Low ambient temperatures also trigger physiological stress, as veins and arteries constrict and blood becomes more viscous, elevating cardiac workload and mirroring some of the cardiovascular strain observed under heat exposure [17]. High temperature anomalies may directly cause heat stress in young children [18]. Indirectly, rising temperatures can increase the prevalence of vector-borne diseases such as malaria and dengue [19], which disproportionately affect children in low-resource settings. Rising average temperatures can have immediate effects, such as those seen during natural disasters and extreme events like floods, hurricanes, droughts, and heat waves, as well as gradual impacts over time, including reduced water availability, soil degradation, loss of arable land, and heightened pollution [20,21]. Additionally, temperature extremes can undermine the well-being and productivity of adults within the household, reducing their capacity to provide adequate care, nutrition, and supervision for children [22,23].

Burkina Faso and Kenya serve as valuable case studies for examining the relationship between child malnutrition and temperature extremes due to their distinct climates and socio-economic contexts. Burkina Faso, located in the Sahel region, frequently experiences extreme heat and prolonged dry spells, which negatively affect food security and contribute to malnutrition, particularly in rural areas reliant on subsistence agriculture [24]. Kenya, with its more varied climate, faces both droughts in arid regions and flooding in more humid areas, exacerbating food insecurity and malnutrition in vulnerable populations [25,26]. Both countries suffer from high rates of child malnutrition, making them ideal for studying how temperature extremes impact nutrition. This was showcased in the Food Security Information Network (2024) which found that about 25–50% of Kenya’s population was experiencing acute food insecurity and Burkina Faso’s population was estimated to be 10–25% acutely food insecure [27].

### 1.1. Previous Research

Historically, extreme variations in temperatures have broadly proven to impact child health [28,29,30]. Policy makers concerned about extreme temperature variation focus on several interrelated health, social, infrastructure and governance issues. Key areas of concern include increased risk of heat- and cold-related illness [31] and nutritional and food security impacts (e.g., disrupted agriculture and food systems can reduce household income and food access) [32]. Within the current era of global climate change, countries will experience heatwaves and cold shocks, both often being defined by either exceeding the average temperature of a region or falling significantly below.

#### 1.1.1. Heat

A previous study based on data from Demographic and Health Surveys (DHS) across sub-Saharan Africa including Kenya and Burkina Faso findings revealed that monthly mean daytime land surface temperatures exceeding 35 °C were significantly associated with increased odds of wasting, underweight, and concurrent stunting with wasting [33]. Interestingly, ref. [34] showed that the odds of stunting alone were lower at these extreme temperatures compared to temperatures below 30 °C. These findings underscore the broader implications of rising global temperatures for population health, particularly in vulnerable regions such as sub-Saharan Africa, where child malnutrition remains alarmingly prevalent [18,35,36,37,38]. Recent modeling studies suggest that climate change-induced heat impacts causing extreme temperatures disproportionately impact socioeconomically disadvantaged groups [39,40], who are likely to experience severe temperature extremes sooner than wealthier populations [41,42]. This trend is expected to continue under current levels of greenhouse gas emissions [43,44]. For instance, ref. [45] projected that if current warming trends persist, the annual frequency of hot days in low-income countries could triple within two decades, compared to the 1961–1990 baseline. The prevalence of undernutrition among children under five in Sub-Saharan Africa (SSA) is estimated at 34%, affecting nearly one-third of the region’s young population [46].

#### 1.1.2. Cold

Much of the existing literature overlooks the health impacts of cold extremes in the Sub-Saharan African context, a gap of particular concern given the heightened vulnerability of these populations to climate stress [47,48]. Existing studies tend to focus on Northern Africa and the country of South Africa, often emphasizing the role of elevation [49,50,51]. The impact of cold temperature events on child health remains understudied. One study [52] found that in Tanzania, for children under five born between 2003 and 2015, a 10-percentage-point increase in days with temperatures below 15 °C (one standard deviation below the long-term average) was associated with a 2.0 and 1.4 percentage-point increase in the probability of stunting and severe stunting, respectively—equivalent to 5.5% and 9.7% of the mean rates. The effects of cold waves appeared more pronounced in warmer regions than in colder areas [53,54].

## 2. Materials and Methods

Here, we use the 2022 Demographic and Health Survey data from Kenya and 2021 in Burkina Faso, along with temperature data from the Climate Hazards Center InfraRed Temperature with Station data (CHIRTS) five-kilometer global dataset. Below we describe the data and methods used.

### 2.1. Data

This study focuses on Kenya and Burkina Faso, utilizing the most recent Demographic and Health Surveys (DHS) rounds conducted in 2022 and 2021, respectively. The analysis centers on the child recode file, which provides detailed information on child health outcomes. Specifically, this study examines stunting, two critical indicators of child malnutrition.

In the 2022 Kenya Demographic and Health Survey (KDHS) [55], the sample included 1692 clusters, with 25 households selected per cluster, totaling 42,300 households. However, some clusters contained fewer than 25 households; in such cases, all households within those clusters were selected. For the 2021 Burkina Faso Demographic and Health Survey (DHS) [56], included 6344 children from 600 sampled clusters from a total sample size of 13,251 households. Due to differences in sample size and data quality between the two countries’ DHS surveys, the analytic sample was reduced by excluding non-relevant demographic groups (children under 24 months of age and adults) and observations with missing values (KNBS and ICF. 2023, INSD and ICF. 2023).

Childhood malnutrition was assessed using stunting among children under five. Stunting was selected over other indicators such as wasting or underweight, which are sensitive to short-term factors like illness or inadequate feeding. In contrast, stunting provides a more stable measure of chronic malnutrition, reflecting long-term deficits in linear growth [9].

### 2.2. Methods

**Figure 1:**
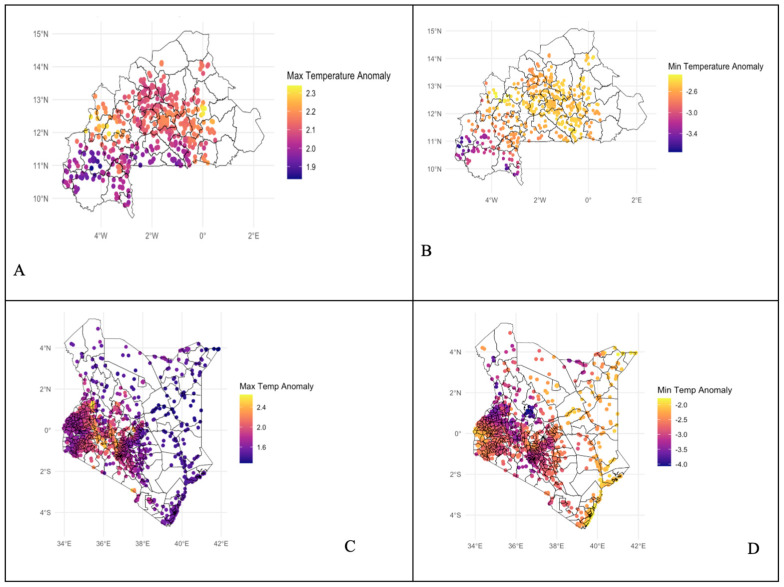
(**A**) Observations in Burkina Faso average hot temperature anomalies positive anomalies in 2016–2021. (**B**) Observations in Burkina Faso cold temperature anomalies, with negative anomalies in 2016–2021. (**C**) Observations in Kenya hot temperature anomalies, with positive anomalies in 2017–2022. (**D**) Observations in Kenya cold temperature anomalies, with negative anomalies in 2017–2022.

Cold snaps and heatwaves have been defined based on the duration of days in which temperatures fall below or rise above the average temperature for a given region [57]. In this study, to account for spatial heterogeneity, heatwaves and cold snaps observed in Figure 1 will be defined relative to the average temperature patterns specific to each case study country. Environmental exposures have been determined by aligning temperature increases or decreases with the child’s age and the timing of these climatic events, capturing the relevant exposure window up to the child’s age at the time of the survey. Heatwave intensity has been a stronger predictor of heatwave-related mortality than duration. According to the literature, hot days are typically defined as temperatures reaching 30 degrees Celsius or exceeding the 98th percentile for at least three consecutive days [58,59]. Cold weather shocks have been defined as a minimum temperature falling more than one standard deviation below the long-term average [52]. While the incidence of environmental heat and cold exposure (EHCE) generally declined across most regions between 1990 and 2019, some areas experienced an increase. The most significant rise occurred in Southern sub-Saharan Africa, followed by Eastern sub-Saharan Africa [60,61,62].

To capture climate anomalies experienced by children aged 24 to 59 months, we calculated standardized z-scores for both maximum and minimum temperature values. Specifically, we utilized the CHIRTSmax ERA5 [63] dataset for daily maximum temperatures. These climate time series were spatially linked to each DHS cluster by matching geolocated DHS cluster coordinates with the corresponding grid cells from the CHIRTSmax datasets. This approach allowed us to assess how cumulative exposure to temperature anomalies may be associated with child stunting risk in different regions. Age stratification is conducted by isolating children into a group of 24–59 months of age. This approach facilitates a more precise analysis of how exposure to climate extremes may influence stunting and outcomes [64]. It should noted that these DHS clusters and data are spatially buffered to protect participant confidentiality while still allowing for localized analyses.

For each cluster, we extracted the relevant time window representing the 24 months from birth to age 24 months—an age range that encompasses critical early-life exposure to climate variability. We then computed monthly mean anomalies relative to short-term historical averages relevant to the children’s age range and transformed these values into z-scores. This standardization allowed us to identify periods of unusually high or low temperatures relative to local norms, enabling consistent comparisons across diverse ecological zones [65]. These temperature z-scores served as continuous indicators of climate extremes, with higher positive values reflecting hotter or drier-than-normal conditions.

### 2.3. Model Specification

A multilevel logistic regression model was employed [66], accounting for the nested structure of children within DHS clusters by including random intercepts. The design allows for the estimation of associations between environmental exposures and child health outcomes, while adjusting for key covariates such as age, sex, electricity access, water source, sanitation, and urban/rural residence [67]. Temperature anomalies were calculated using the cumulative maximum values within the maximum average temperatures across the clusters as well as using the minimum values within the minimum average temperatures [65]. We converted all household relevant variables into binary values in order to indicate the presence or absence of factors such as electricity, approved water source, wealth and sanitation (Table 1).

## 3. Results

We used a specified model assessing the effects of extreme maximum and minimum temperature exposure on the probability of stunting, with the objective of determining whether particular climatic conditions are associated with increases in the prevalence of acute malnutrition. This modeling framework enabled us to evaluate how deviations at both ends of the temperature spectrum influence child nutritional outcomes and to identify potential thresholds beyond which climate stressors may significantly elevate health risks.

**Figure 2:**
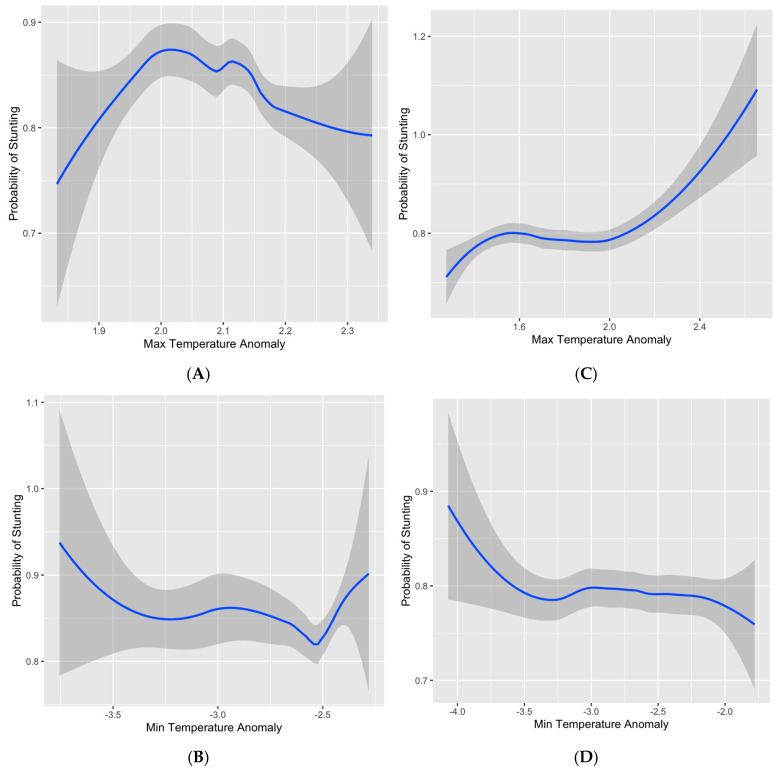
Modeled relationships between temperature anomalies and the probability of child stunting (ages 24–59 months), showing (**A**) Burkina Faso average maximum temperature anomalies (2016–2021), (**B**) Burkina Faso average minimum temperature anomalies (2016–2021), (**C**) Kenya average maximum temperature anomalies (2017–2022), and (**D**) Kenya average minimum temperature anomalies (2017–2022).

Here, we examine the relationship between both cold and hot temperature extremes and child nutrition health outcomes in low-income settings [68]. Specifically, we investigate whether such events, including heat and cold anomalies, are associated with increased risk of child undernutrition by comparing the impact of these anomalies across these two diverse African countries with differing climatic and economic contexts. (Figure 2) presents relationships between temperature anomalies exposure and the probability of stunting among 25–59 months children in Burkina Faso (A and B) and 25–59 months in Kenya (C and D). For Burkina Faso, Panel A shows that the probability of stunting initially increases with maximum temperature anomalies before leveling off and declining at higher anomaly values which may reflect regional survivalhood and adaptability [69,70], while Panel B indicates that minimum temperature anomalies are generally associated with a lower probability of stunting, with a slight increase at the most extreme cold deviations. In Kenya, Panel C demonstrates a strong positive association between maximum temperature anomalies and stunting probability, particularly at higher anomaly values, whereas Panel D shows that higher minimum temperature anomalies are associated with a gradual decrease in the probability of stunting as well as the shaded areas indicating greater uncertainty at the extremes of observed temperature anomalies.

Given the likelihood that the most pronounced effects occur at the extremes of the temperature distribution, we employ a generalized linear multivariate regression model to capture and quantify these relationships. This approach allows us to examine not only the direct association between temperature anomalies and child stunting, but also the interaction effects between climatic variables and key household factors such as access to safe water, electricity, and improved sanitation that may either exacerbate or mitigate the observed impacts. By modeling these interactions, we aim to better understand how environmental and socio-economic determinants jointly influence stunting risk.

Results from exposure to climate anomalies are summarized in (Table 2) and highlight how environmental and socioeconomic factors shape the risk of stunting among children aged 24–59 months. Cold anomalies (minimum temperature) were marginally associated with higher odds of stunting in both Burkina Faso (BF; OR = 2.14, *p* < 0.1) and Kenya (KE; OR = 1.20, *p* < 0.1), potentially reflecting cooler, wetter periods that increase diarrheal disease or malaria transmission and disrupt food availability. In contrast, heat anomalies (maximum temperature) were associated with markedly lower odds of stunting in BF (OR = 0.08, *p* < 0.05) but significantly higher odds in KE (OR = 2.34, *p* < 0.001), suggesting that in BF extreme heat may reduce pathogen survival through drier conditions, whereas in KE it may exacerbate food insecurity, water scarcity, or heat stress that negatively affect child growth. Electricity access was protective in both countries (BF OR = 0.61, *p* < 0.05; KE OR = 0.71, *p* < 0.001), likely reflecting improved socioeconomic conditions, safer food storage, and healthier household environments. Access to an improved water source was not significant in BF but was associated with lower odds of stunting in KE (OR = 0.80, *p* < 0.05), likely reflecting differences in water contamination risk. Older child age was consistently protective (BF OR = 0.83, *p* < 0.05; KE OR = 0.75, *p* < 0.001), consistent with evidence that the highest risk of growth faltering occurs in early life. Male children had slightly lower odds of stunting in KE (OR = 0.86, *p* < 0.05), suggesting potential gender differences in feeding or care practices. Urban residence was not protective in BF (OR = 1.87, *p* < 0.01) but not significant in KE, pointing to stronger potential of urban poverty in BF. Improved sanitation was significantly protective in KE (OR = 0.64, *p* < 0.001) but not in BF, and wealth showed a strong protective association in KE (OR = 0.58, *p* < 0.001) but no significant effect in BF, indicating that household resources may be more important for mitigating stunting in KE. Taken together, these findings underscore that child stunting is shaped by an interaction of climatic shocks, infectious disease exposure, and household-level socioeconomic conditions, reinforcing the need for locally tailored nutrition and climate resilience interventions.

**Figure 3:**
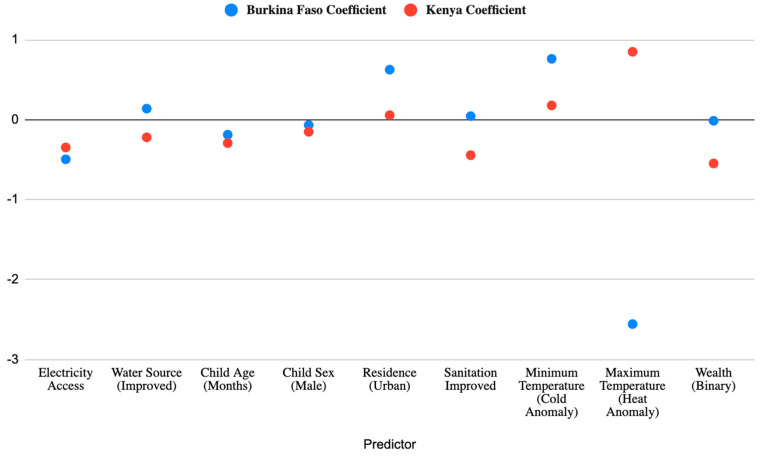
Comparative Analysis of Coefficients of Kenya and Burkina Faso. Logistic regression coefficients show both shared and divergent determinants of child stunting across the two countries.

Shown in Figure 3 is a divergence is seen in the climate-related predictors. In Kenya, higher maximum temperature anomalies are associated with increased stunting, suggesting that heat stress may harm child growth. Conversely, in Burkina Faso, the relationship is reversed: higher maximum temperatures appear to reduce stunting odds, a counterintuitive result that may reflect unobserved confounders (Table 2). Minimum temperature anomalies are marginally significant and positive in both contexts, hinting at a potential need for researchers to look at adaptation capacity for colder temperatures or extreme precipitation events in increasing nutritional risk within the African context.

## 4. Discussion

The relationship between stunting and climate is influenced by factors such as the intensity and timing of climate-related shocks, along with local geographical conditions and the characteristics of both the child and household [71,72]. The findings from these models underscore the importance of country-specific policy approaches, as the household-level factors that influence child stunting and growth vary across socio-economic and environmental contexts. What mitigates stunting in one setting may be less relevant or even counterintuitive in another. Specifically, this study explored how socioeconomic factors, such as household wealth and access to infrastructure like electricity and safe water, may buffer or exacerbate these effects. Comparative analysis (seen in Figure 3 and Table 3) allows for the incorporation of key features identified in previous multilevel studies within a single investigation of two countries exhibiting elevated rates of child malnutrition. By focusing on two climate and health contexts, this study also seeks to identify potential pathways for intervention and policy development [25,73]. Lastly, it considers whether there are differential impacts between urban and rural settings, aiming to understand how location-based disparities may influence children’s vulnerability to climate-related nutritional stress.

Mitigating malnutrition stands as a paramount global health imperative, resonating with various objectives delineated within the United Nations’ Sustainable Development Goals (SDGs) particularly Goal 2 “Zero Hunger” which highlights the role of climate shocks as well as the rising cost of living. As infrastructure evolves and environmental conditions shift, the opportunity to prevent child malnutrition lies in forward-thinking investments in water, sanitation and hygiene (WASH) and comprehensive preparedness efforts [74,75]. Demographic and Health Survey (DHS) data continue to shed light on potential household-level interventions that may improve child health outcomes, particularly for children under five. One such intervention is investment in electricity access, especially renewable energy options which are often more cost-effective and sustainable in low-resource settings. Though this variable was statistically significant in one case study country, it highlights the need for development-oriented interventions. Limited access to electricity has been linked to reduced life expectancy, higher infant mortality rates, increased per capita health expenditures, and overall poorer health outcomes [76]. Additionally, the divide between urban and rural areas underscores enduring wealth inequalities that shape household living conditions and, in turn, children’s early-life exposures [76,77,78]. Beyond financial disparities, geographic differences are evident, with the model’s results indicating that rural disadvantage is more pronounced in Burkina Faso than in Kenya. Understanding that both heatwaves and cold snaps can shape a child’s life early on makes them both important to consider when observing future climate adaptation and/or mitigation strategies to implement.

The observed reduction in the probability of stunting across countries particularly with the child sex variable underscores the nuanced influence of gender dynamics on child health outcomes. This pattern is consistent with findings from prior studies. For instance, ref. [79] reported that in Burkina Faso, the prevalence of underweight and stunting among children is significantly shaped by demographic factors, particularly the sex of the child. Their study further identified critical determinants of undernutrition, including ethnic background, household economic status, the number of children under five residing in the household, and proximity to health facilities. These variables collectively reflect entrenched socio-economic and structural barriers to adequate child nutrition. Complementing these findings [80], in a comprehensive analysis of Demographic and Health Surveys (DHS) data from 30 sub-Saharan African countries including Kenya demonstrated that women’s socio-demographic profiles and levels of empowerment (measured through decision-making autonomy, attitudes toward domestic violence, and personal experiences of violence) were significantly associated with child nutritional outcomes, specifically stunting and underweight, with results statistically significant at *p* < 0.001.

Understanding resource and infrastructure accessibility at the household level informs how climate anomalies may impact health outcomes within a household [81,82]. One study highlighted the complex relationship between urbanization and childhood health outcomes in Africa [83]. While it is widely recognized that children in urban regions generally experience better health outcomes, the degree of urbanization in a given region plays a critical role. It has been reported that average energy poverty levels remain high globally, with prevalence rates of 54% in Sub-Saharan Africa, 41% in South Asia, and 23% in Latin America and the Caribbean [84]. Their study also demonstrated that energy poverty undermines childhood development by affecting living standards and child health. In Kenya, energy poverty is a persistent challenge, particularly in rural areas. Highlighting this distinction a study found that its negative health impacts are more pronounced in rural households than in urban ones [85]. Similarly, in Burkina Faso, a study identified limited access to modern cooking fuels as a key driver of household energy deprivation, which disproportionately affects girls’ school attendance compared to boys [86]. These infrastructural influences are also true when considering sanitation and water accessibility as some research has found it to have detrimental effects on child growth and development from sustained exposure to enteric pathogens but also due to wider social and economic mechanisms [87].

Globally, the pediatric health implications of exposure to extreme temperatures continue to be observed throughout the scientific literature. This study contributes to a growing body of evidence demonstrates that children are uniquely vulnerable to these conditions, as one scoping review highlighting a broad range of adverse outcomes, including preterm birth, low birth weight, emergency visits for asthma, heat-related illnesses, and impaired school performance, underscoring the pervasive impacts of rising temperatures on child health and development [88]. Complementing this, a narrative review of pediatric and perinatal outcomes found that maternal heat exposure elevates risks of pre-eclampsia, gestational diabetes, and preterm birth, while children themselves experience heightened risks of heatstroke, dehydration, renal impairment, respiratory and gastrointestinal infections, as well as cognitive and mental health disturbances [89]. Systematic reviews further emphasize the association between heatwave exposure in infants and young children and increased incidence of renal disease, respiratory conditions, electrolyte imbalance, and fever [90]. Expanding on these findings, a broader scoping review revealed consistent correlations between high ambient temperatures and a spectrum of pediatric morbidities, including heat-related illness, dehydration, gastrointestinal symptoms, infectious diseases, asthma, and injuries [91]. Collectively, this literature points to extreme heat and cold as a multifaceted and growing threat to child health across developmental stages.

Like all studies using the DHS data, there are limitations to our approach. This study uses household and individual response information available in the surveys for the two countries. However, the surveys may lack direct contextual information which could help the model identify the cause of nutrition deficits during periods of temperature anomalies. For example, availability of electricity may not mean that the household uses it to improve its adaptation to heat or cold. To reduce potential confusion due to the spatial offset of cluster coordinates, we use best practices to match the cluster to the climate information, as set out in [65,92].

## 5. Conclusions

In conclusion, this study highlights several factors that may be associated with stunting among children aged 24 to 59 months in Burkina Faso and Kenya. Exposure to extreme heat anomalies was observed to be linked with higher stunting risk in both contexts, aligning with growing evidence that climate-related heat events can have important implications for child nutrition. Socio-demographic and infrastructural characteristics such as urban versus rural residence, child sex, access to electricity, and availability of safe drinking water also showed variable associations with nutritional outcomes, reflecting underlying structural inequities. These findings should be interpreted cautiously given the context-specific sample and the cross-sectional design, which limits causal inference. While the results suggest that improving household and community infrastructure, expanding access to safe water and electricity, and addressing broader socio-economic disparities could potentially reduce child stunting, any policy or programmatic recommendations are necessarily tentative. Future research employing longitudinal designs and larger, more diverse populations is needed to confirm these associations and to better guide interventions. Nonetheless, these determinants underscore persistent structural inequities that, if addressed through targeted policy and programmatic efforts, could contribute to meaningful improvements in child nutrition over time.

## Figures and Tables

**Table 1:** Description of socio-economic and climate-related variables used in the analysis, including variable names, measurement scales, and definitions.

Variable	Definition
Child Age (Months)	Age of the child in months at the time of survey.
Child Sex	Biological sex of the child (male/female).
Electricity Access	The household has access to electricity (binary: yes/no).
Maximum Temperature (Tmax)	CHIRTS dataset: Daily maximum temperature, averaged for the exposure window (e.g., 59 months before survey).
Minimum Temperature (Tmin)	CHIRTS dataset: Daily minimum temperature, averaged for the exposure window (e.g., 59 months before survey).
Residence (Urban)	Urban versus rural place of residence (binary: urban = 1, rural = 0).
Sanitation Improved	The household uses an improved sanitation facility per WHO/UNICEF Joint Monitoring Programme definition (binary: yes/no).
Water Source	The household uses an improved drinking water source per WHO/UNICEF definition (binary: yes/no).
Wealth (Binary)	Household wealth index categorized as high (1) versus low (0) wealth, based on DHS quintiles.

**Table 2:** Multilevel logistic regression results for the association between socio-economic factors, climatic anomalies, and the odds of stunting among children 24–59 months in Burkina Faso (n = 1384) and children 24–59 months in Kenya (n = 5097). Estimates are reported as log-odds coefficients, odds ratios (OR), and 95% confidence intervals (CI).

Predictor	Burkina Faso Coefficient	BF OR	95% CI (BF)	Kenya Coefficient	Kenya Coefficient	KE OR	95% CI (KE)
**Electricity Access**	−0.496 *	0.609	[0.396, 0.937]	−0.347 ***	−0.347 ***	0.707	[0.594, 0.842]
**Water Source (Improved)**	0.138	1.147	[0.798, 1.648]	−0.221 *	−0.221 *	0.802	[0.671, 0.958]
**Child Age (Months)**	−0.188 *	0.829	[0.688, 0.999]	−0.293 ***	−0.293 ***	0.746	[0.682, 0.815]
**Child Sex (Male)**	−0.067	0.935	[0.701, 1.247]	−0.152 *	−0.152 *	0.859	[0.747, 0.988]
**Residence (Urban)**	0.625 **	1.867	[1.207, 2.887]	0.056	0.056	1.057	[0.888, 1.258]
**Sanitation Improved**	0.044	1.045	[0.717, 1.524]	−0.444 ***	−0.444 ***	0.641	[0.543, 0.758]
**Minimum Temperature (Cold Anomaly)**	0.761 .	2.141	[0.930, 4.925]	0.178 .	0.178 .	1.195	[0.996, 1.433]
**Maximum Temperature (Heat Anomaly)**	−2.557 *	0.077	[0.006, 0.889]	0.849 ***	0.849 ***	2.338	[1.637, 3.340]
**Wealth (Binary)**	−0.014	0.986	[0.659, 1.473]	−0.548 ***	−0.548 ***	0.578	[0.460, 0.726]

Note. Models include random intercepts for DHS regions to account for geographic clustering. Significance codes: *** *p* < 0.001, ** *p* < 0.01, * *p* < 0.05, . *p* < 0.10.

**Table 3:** Fitness of Models from model results. Cluster-level variance was negligible in Burkina Faso but present in Kenya, with differences in AIC, BIC, and log-likelihood reflecting the larger Kenyan sample.

Country	Random Intercept Variance	AIC	BIC	Log Likelihood	Observations	Clusters
Burkina Faso	~0 (1.41 × 10^−13^)	1242.3	1294.6	–611.1	1384	380
Kenya	0.1199	5035.2	5100.5	–2507.6	5097	1523

## Data Availability

The data underlying this study are derived from the Demographic and Health Surveys (DHS) Program, implemented by ICF International and funded primarily by the United States Agency for International Development (USAID), with additional contributions from international partners including UNICEF, UNFPA, WHO, and UNAIDS. Access to DHS datasets is restricted but can be obtained free of charge upon formal registration and approval of a data request through the DHS Program website (https://dhsprogram.com/data/) (accessed on 20 August 2024).
